# Stimulating the Uptake of Preconception Care by Women With a Vulnerable Health Status Through mHealth App–Based Nudging (Pregnant Faster): Cocreation Design and Protocol for a Cohort Study

**DOI:** 10.2196/45293

**Published:** 2023-08-09

**Authors:** Sharissa M Smith, Babette Bais, Hafez Ismaili M'hamdi, Maartje H N Schermer, Regine P M Steegers-Theunissen

**Affiliations:** 1 Department of Obstetrics and Gynecology Erasmus University Medical Center Rotterdam Netherlands; 2 Department of Medical Ethics, Philosophy and History of Medicine Erasmus University Medical Center Rotterdam Netherlands

**Keywords:** preconception care, periconception, health inequality, socioeconomic status, lifestyle, ethics, mobile health, mHealth, mental stressor, pregnancy, mobile phone

## Abstract

**Background:**

Women with a low socioeconomic status often have a vulnerable health status due to an accumulation of health-deteriorating factors such as poor lifestyle behaviors, including inadequate nutrition, mental stressors, and impaired health literacy and agency, which puts them at an unnecessary high risk of adverse pregnancy outcomes. Adequately preparing for pregnancy through preconception care (PCC) uptake and lifestyle improvement can improve these outcomes. We hypothesize that *nudging* is a successful way of encouraging engagement in PCC. A nudge is a behavioral intervention that changes choice behavior through influencing incentives. The mobile health (mHealth) app–based loyalty program *Pregnant Faster* aims to reward women in an ethically justified way and nudges to engage in pregnancy preparation by visiting a PCC consultation.

**Objective:**

Here, we first describe the process of the cocreation of the mHealth app *Pregnant Faster* that aims to increase engagement in pregnancy preparation by women with a vulnerable health status. Second, we describe the cohort study design to assess the feasibility of *Pregnant Faster*.

**Methods:**

The content of the app is based on the eHealth lifestyle coaching program Smarter Pregnancy, which has proven to be effective in ameliorating preconceptional lifestyle behaviors (folic acid, vegetables, fruits, smoking, and alcohol) and an interview study pertaining to the preferences of the target group with regard to an mHealth app stimulating PCC uptake. For moral guidance on the design, an ethical framework was developed based on the bioethical principles of Beauchamp and Childress. The app was further developed through iterative cocreation with the target group and health care providers. For 4 weeks, participants will engage with *Pregnant Faster*, during which opportunities will arise to earn *coins* such as reading informative blogs and registering for a PCC consultation. Coins can be spent on small fun rewards, such as folic acid, fruits, and mascara. *Pregnant Faster*’s feasibility will be tested in a study including 40 women aged 18 to 45 years, who are preconceptional or <8 weeks pregnant, with a low educational level, and living in a deprived neighborhood. The latter 2 factors will serve as a proxy of a low socioeconomic status. Recruitment will take place through flyers, social media, and health care practices. After finalization, participants will evaluate the app through the “mHealth App Usability Questionnaire” and additional interviews or questionnaires.

**Results:**

Results are expected to be published by December 2023.

**Conclusions:**

*Pregnant Faster* has been designed through iterative cocreation with the target group and health care professionals. With the designed study, we will test *Pregnant Faster*’s feasibility. If overall user satisfaction and PCC uptake is achieved, the app will be further developed and the cohort will be continued with an additional 400 inclusions to establish effectiveness.

**International Registered Report Identifier (IRRID):**

DERR1-10.2196/45293

## Introduction

### Background

**Figure 1:**
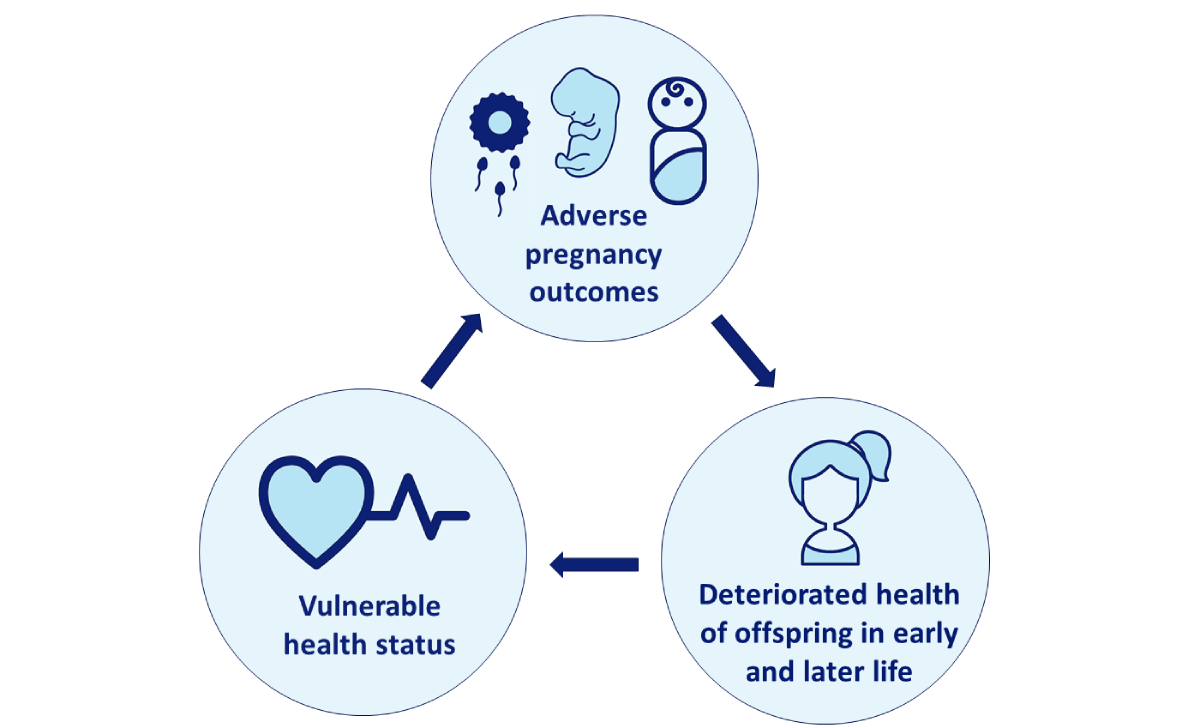
Generational impact of the vulnerable health status in women with a low socioeconomic status.

Although decreasing, perinatal morbidity and mortality rates are still relatively high in the Netherlands, especially among women who live in deprived neighborhoods and have a low socioeconomic status (SES) [1-3]. These women, who represent up to 45% of the total population in the 4 largest cities in the Netherlands [4], are more likely to have adverse pregnancy outcomes based on the accumulation of health-deteriorating risk factors such as poor lifestyle behaviors (eg, smoking, alcohol consumption, and sedentary lifestyle), inadequate nutrition (consumption of unhealthy food or insufficient intake of healthy food), mental stressors, and impaired health literacy and agency [5-7]. These risk factors, some of which are modifiable, can detrimentally influence the pregnancy course and outcome in the periconceptional phase comprising the preconceptional period of gametogenesis and the first 3 months of embryogenesis and placentation [8]. Complications that occur later in pregnancy are also more common among these women, such as preeclampsia and low birthweight [9-11]. These outcomes result in increased perinatal morbidity and mortality that has its origin in the periconceptional period. Furthermore, there is an increasing body of evidence showing that these pregnancy complications increase the risk of noncommunicable diseases in mothers and their infants during their life course, such as obesity, cardiovascular disease, and diabetes [12,13]. Therefore, we categorized these women with a low SES as women with a vulnerable health status. In short, the vulnerable health status of women with a low SES leads to an increased risk of adverse pregnancy outcomes, which in turn leads to deterioration of the offspring’s health, causing them to have a vulnerable health status as well, impacting the health and lives of generations (Figure 1).

Most clinical manifestations of adverse pregnancy outcomes, such as hypertension or fetal growth restriction, are terminal features of a cascade of events that originate in the periconceptional period (ie, the 14 weeks before conception up to the first 10 weeks of pregnancy) [14]. During this period, on which the focus is limited in research as well as in clinical practice, modifiable poor lifestyle behaviors are known to be common risk factors for subfertility and adverse pregnancy course and outcomes [15-17]. For example, ≥80% of women who are contemplating pregnancy have at least one modifiable behavior associated with adverse pregnancy outcomes, such as not taking folic acid supplements, low vegetable and fruit intake, and smoking [18,19].

Preconception care (PCC) focuses on improving women’s health, including lifestyle behaviors, before pregnancy and plays an essential role in encouraging healthy lifestyle behaviors in preparation for pregnancy. Therefore, it reduces the risk of adverse pregnancy outcomes and improves health outcomes in mothers and their children now and later in life [20,21]. PCC is usually delivered face-to-face, for example, at midwifery practices or hospitals. However, web-based PCC has been gaining popularity, which are delivered through video consultations or even combined as blended care [22].

The low uptake of PCC by the general population in the Netherlands is associated with women’s perceived absence of risks and lack of awareness of PCC [23]. The delivery of PCC for women with a vulnerable health status faces even more obstacles, as these women are more difficult to reach and more difficult to motivate to engage in PCC [24]. These obstacles may lead to an ethically unjustifiable inequality in the health outcomes and life prospects of mothers and children from different socioeconomic backgrounds [24].

To encourage pregnancy preparation, our research group developed the web-based mobile health (mHealth) platform *Smarter Pregnancy* [19], an internet-based, tailored coaching platform that supports women who wish to become pregnant (and possibly their partners) to adopt healthy lifestyle behaviors (such as folic acid supplement use, adequate intake of vegetables and fruits, and cessation of smoking and alcohol consumption) before and during pregnancy. Previous research on the effectiveness of *Smarter Pregnancy* has shown that these healthy behaviors increase by at least 30% and that the chance of an ongoing pregnancy after fertility treatment increases by more than 50% [19,25,26]. Furthermore, *Smarter Pregnancy* has proven to be effective and highly valued by women from deprived neighborhoods [27]. This shows the potential of tailor-made mHealth for women with a vulnerable health status and underlines the importance of customizing an intervention that aims to overcome the obstacles associated with reaching and motivating women with a vulnerable health status to engage in PCC.

Changing behavior and lifestyle habits is challenging for all but even more so for women with a low SES, as they often endure stressful situations and deprived circumstances [28,29]. Therefore, it is advisable to offer this group more support in changing their behavior, which also follows the demands of social justice [30]. One way to support behavioral change is through *nudging*. Nudges are a class of interventions that change people’s choice behavior by influencing their incentives, thereby stimulating beneficial, healthy behaviors that individuals themselves value [31]. This could be, for instance, offering fruit instead of chocolate bars at checkout counters to increase fruit consumption and lower refined sugar intake. Previous research has shown that nudging is a promising intervention to encourage the improvement of healthy eating behaviors [32]. Another example of nudging is loyalty programs, in which people can gather rewards by making healthy choices. Nudging via a smartphone app, especially designed for women with a vulnerable health status, may be very suitable to motivate them to engage in PCC due to the current wide availability of smartphones, as well as among women with a vulnerable health status.

### Hypothesis

We hypothesized that nudging via the app *Pregnant Faster* is a feasible way to encourage pregnancy preparation and increase PCC uptake in women with an SES-related vulnerable health status. We expect that this will eventually lead to improved maternal health and pregnancy outcomes as well as improved health of the offspring in early and later life.

### Aims

In this paper, we aimed to describe in detail the cocreation design process of the mHealth app *Pregnant Faster*, a loyalty program embedded in an app, based on the tailored mHealth lifestyle coaching platform *Smarter Pregnancy.* Furthermore, we aimed to develop a cohort study protocol to test the feasibility of *Pregnant Faster*, comprising overall user satisfaction; the number of booked and visited PCC consultations; and the course of practical conduction with regard to the inclusion process, reward allocation, and finalization. The general goal of *Pregnant Faster* is to encourage women with a vulnerable health status, in an ethically justified way, to adequately prepare for pregnancy by visiting a PCC consultation.

If the results of this study indicate that *Pregnant Faster* is appreciated by the participants and stimulates the uptake of PCC, we aim to continue this proposed study with an updated version of *Pregnant Faster* and an extended cohort, to establish the effectiveness of increasing the uptake of PCC in women with an SES-related vulnerable health status.

Our ultimate goal is to contribute to the reduction of inequalities in the health of women before and during pregnancy by (1) creating awareness regarding women’s own influence on the course of their pregnancy and an increased uptake of PCC, (2) reducing perinatal morbidity and mortality, and (3) improving the long-term physical and mental health of mothers and their children.

## Methods

### The Cocreation Process

#### Preparation

The preparation of the design process of *Pregnant Faster* can be subsumed under 3 pillars. First, we have developed an ethical framework to assess the ethical justifiability of developing a loyalty program to stimulate PCC uptake among women with a vulnerable health status. An ethical framework can be viewed as a guideline that offers guidance on the moral permissibility of an intervention and provides suggestions on how to avoid ethical pitfalls, such as exposing susceptible participants to stigmatization based on their SES. This framework was developed by performing a literature search to identify records broadly relevant to the subject of using nudges to stimulate healthy lifestyle behaviors and extracting ethical dilemmas and pitfalls from these records. On the basis of these extracted dilemmas and pitfalls, criteria were set up to which an incentive-based intervention encouraging pregnancy preparation in susceptible women must comply to be morally justifiable. These criteria were subdivided under the 4 ethical principles of Beauchamp and Childress [33]: (1) respect for autonomy, (2) beneficence, (3) nonmaleficence, and (4) justice. By addressing all 4 principles, an attempt was made to address as many ethical pitfalls as possible without overlooking any major aspects. This *principlist* approach is ideally suited for the topic of nudging women with an SES-related vulnerable health status because it allows for balancing between the duty to improve their and their children’s health and well-being and the duty to respect them and treat them as free and equal persons.

Second, we conducted semistructured interviews with 15 women from the target group, which allowed us to gain insight into how they regard loyalty programs; which type of loyalty program they prefer; and which rewards are considered most legitimate, attractive, and effective [34]. The interviews were analyzed using a framework approach and thematic content analyses of five themes: (1) “usefulness of an app as an integral information source,” (2) “permissibility and effects of offering rewards,” (3) “preferences regarding content,” (4) “preferences regarding the type of rewards and system of allocation,” and (5) “barriers.” Involving the target group in the design of the app, thereby respecting their autonomy and preferences, is an imperative criterion of the ethical framework.

Third, we based the lifestyle-related content of the app on the tailored mHealth lifestyle coaching platform *Smarter Pregnancy. Smarter Pregnancy* has previously proven to be effective in ameliorating preconceptional health and lifestyle behaviors and is based on over 30 years of research and the expertise of this research group regarding the influence of nutrition and lifestyle on reproduction and pregnancy course and outcome [25]. The main focus of *Smarter Pregnancy* lies in the amelioration of nutrition, including folic acid supplement use, and supporting healthy lifestyle behaviors such as reaching a healthy weight, smoking and alcohol cessation, and sufficient physical activity.

The knowledge gained through these 3 pillars was combined into a first design of the app *Pregnant Faster*. The choice to develop this loyalty program as a nudge was based on the fact that women are known to wish for a healthy baby when they want to become pregnant [23,35]. Thus, the definition of nudging is that a nudge always aims to help the *nudgee* reach a goal they themselves wish for and value [31]. In addition, a nudge may not harm the nudgee. This description complies with the aforementioned biomedical ethical principles.

#### Iterative Cocreation

**Figure 2:**
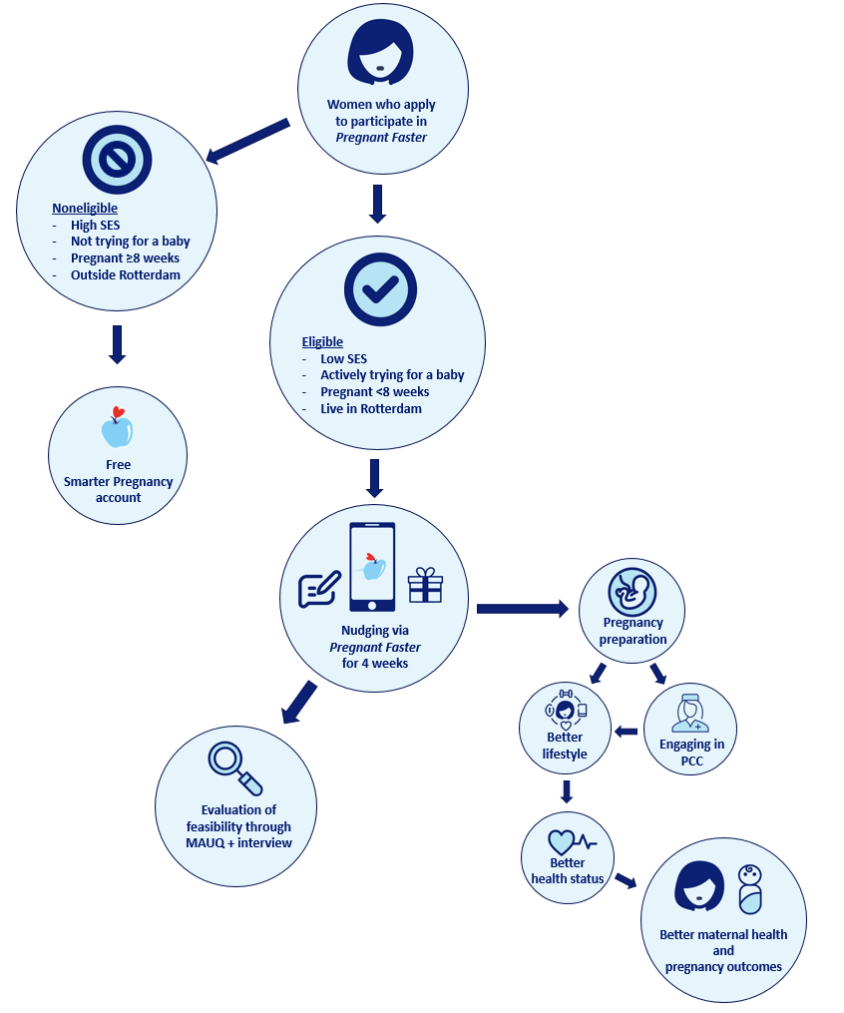
Overview of the intervention. MAUQ: mHealth App Usability Questionnaire; PCC: preconception care; SES: socioeconomic status.

Subsequently, we conducted a strategic work session with professionals in this field (5 midwives, 2 gynecologists, and 2 general practitioners) in which we discussed our findings regarding moral permissibility, the wishes and preferences of the target group, and the first design of the app. The professionals asked to participate were known to practice in low-income areas, providing them with experience in caring for the target group of women with a vulnerable health status. The strategic work session was led by an external communication bureau specialized in transforming these types of sessions and the yielded insights into practical reports. These insights were then implemented in the app, leading to the design of a new and improved version of the app, adhering to the ethical framework. Through an iterative process (Figure 2), adjusted versions of the app were discussed by the communication bureau multiple times with 10 women in the target group who had previously participated in the interview study [34] and the aforementioned professionals, until all were satisfied with the design of the app and the app was considered morally permissible. The resulting app-based nudge *Pregnant Faster* can be viewed as an *umbrella* or a *macro-level* nudge, containing multiple *micro-level nudges*.

### Cohort Study Protocol

#### Overview

This study was set up as an open cohort to reflect the real-life situation, and it aims to assess the feasibility of the mHealth app–based nudge that stimulates PCC uptake in women with an SES-related vulnerable health status.

After inclusion (T0), the women start the intervention, consisting of using the app daily. The total duration of the intervention will be 4 weeks per participant, as we deemed a short period of active engagement more feasible than a longer period and did not expect additional engagement in pregnancy preparation or PCC consultations after 4 weeks of using the app.

**Figure 3:**
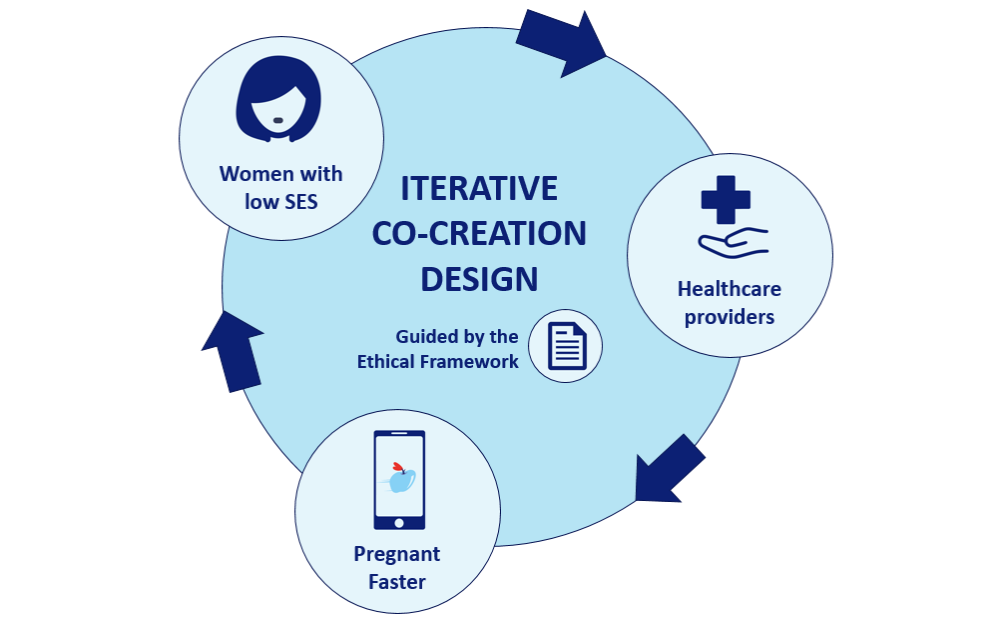
Iterative cocreation of Pregnant Faster. SES: socioeconomic status.

After the intervention (T1), participants fill out the “mHealth App Usability Questionnaire” (MAUQ), assessing the app, as well as partake in an interview in which they may state their opinions regarding the content of the app and how they rate their experience. At T1, we will assess the uptake of PCC as well (Figure 3). The results of this study will provide further insights to enhance the final design of the nudge and the app and can be viewed as another round in the iterative process, implementing the opinions and experiences of the target group into the final design. After this proposed cohort study, we aimed to update *Pregnant Faster* and plan to establish its effectiveness in a cohort of 400 women. Originally, the sample size of 400 was based on a power calculation for a randomized controlled trial with 2 groups using the app: one with and one without the allocation of rewards. However, the choice was made to convert the planned controlled trial to a cohort study due to the nature of this intervention and the possible disappointment women would feel if they would be randomized in a nonreward group, which could lead to disengagement and biased outcomes.

This research group has previously experienced difficulty in including women with a vulnerable health status in an interview study [34]. Although we believe that the nature of the intervention *Pregnant Faster* might encourage more women to participate, we consider these previously obtained experiences. Therefore, for this pilot study, we chose to aim for a sample size of 40 participants (10% of the larger planned cohort study), which we deemed sufficient to provide the first insights supporting further development of *Pregnant Faster*.

#### Participants

To be eligible, participants must meet all the following inclusion criteria: assigned female at birth, 18 to 45 years of age, living in the municipality of Rotterdam (the Netherlands), currently trying to conceive or planning to actively try within 3 months, or pregnant with a gestational age below 7+6 weeks, and having a vulnerable health status. In this study, having a vulnerable health status is defined as having a low SES based on the combination of a low to intermediate educational attainment (prevocational or vocational education) and living in a deprived neighborhood based on the zip code’s median income [36].

A potential participant who meets any of the following exclusion criteria will be excluded from participation in this study: insufficient proficiency in Dutch, not in possession of a smartphone or tablet suitable for the app, living outside of the municipality of Rotterdam, refusal to fill out the questionnaires, or refusal to participate in the interview.

The choice to only include citizens of the municipality of Rotterdam is based on the plan to deliver the rewards per bike, car, or public transport.

#### Recruitment and Inclusion

Women from the target population will be recruited not only through midwifery practices, hospitals, and general practitioner practices but also directly through the distribution of flyers and posters and through social media, specifically Instagram and Facebook. Social media campaigns will be directly targeted at specific areas and populations, which fit our inclusion criteria. Recruitment will take place in collaboration with the Regional Consortium Pregnancy & Childbirth of the Southwest Netherlands, in particular with 5 midwifery practices located in or near deprived neighborhoods of Rotterdam. Women may enroll themselves in the study or they can be enrolled by a health care provider.

When women receive the flyer or are informed about the study by a health care provider, they may fill in their email on the website *Getting Pregnant Faster* [37]. The researcher receives their email address and sends them a screening survey with questions regarding the inclusion criteria. This information will allow for the selection of women who fit the target group. If a potential participant is eligible, the researcher will contact her via telephone and email to provide further information about the study. If the woman is interested in participating, informed consent will be sent via email.

If women are eligible and have signed the informed consent form, they will be asked to download the app and start the intervention. If women are not eligible for participation, they will be provided with a free 26-week subscription to our mHealth coaching platform *Smarter Pregnancy*.

#### Randomization and Blinding

Owing to the nature of the intervention, all participants will receive the same intervention. Therefore, this section is not applicable. Blinding of the participants is not possible. Neither the participants nor the researchers will be blinded.

#### Intervention

The intervention consists of using the app *Pregnant Faster* (Dutch: *Sneller Zwanger*), which can be viewed as a macrolevel nudge containing a multitude of microlevel nudges. All nudges are aimed at encouraging participants to adequately prepare for pregnancy by making healthy nutrition and lifestyle choices and visiting a PCC consultation.

#### Outcome Measures

The main objective of this study design is to establish the feasibility of the app *Pregnant Faster* as a nudge that encourages pregnancy preparation by stimulating the uptake of PCC. Feasibility in this study pertains to overall user satisfaction, the number of booked and visited PCC consultations, and the course of practical conduction with regard to the inclusion process, reward allocation, and finalization. The outcome will be assessed in multiple ways: (1) through the number of women who registered for this study, which indicates the effectiveness of the methods used to reach women; (2) the number of women eligible to participate, which indicates the effectiveness of these methods in reaching the target group; (3) the number of participants who were included and the number who actually started the intervention, which may reveal possible barriers between inclusion and conduction; (4) the number of participants completing the intervention, which offers information on how engaging the app is; and (5) the frequency with which certain rewards are chosen that indicates which rewards are deemed the most desirable and motivating. Furthermore, the usability of the app will be assessed using the MAUQ, a validated 18-item questionnaire on user satisfaction, information arrangement, and usefulness based on a 7-point Likert scale (strongly disagree, disagree, somewhat disagree, neither agree nor disagree, somewhat agree, agree, and strongly agree) as presented in Multimedia Appendix 1 [38].

To further investigate how participants have experienced using the app, the first 10 participants will be interviewed in a semistructured manner to explore the answers they give on questions such as how often they opened the app and if this was according to how often they would have liked to open the app. A topic list and additional information on this interview is present in Multimedia Appendix 2. This topic list and participants’ answers will be converted into a questionnaire offered to the remaining 30 participants. To include the first 10 participants in this questionnaire, the first author will fill in the questionnaire based on the participants’ answers in the interview. The MAUQ and the interview or additional questionnaire will be offered 4 weeks after a participant has started the intervention (T1).

The number of PCC consultation requests and confirmed visits will be registered during the study period and in the aforementioned questionnaires, women will be asked why they registered (or did not register) for a PCC consultation and if they changed their lifestyle behaviors due to using the app. Descriptive statistics will be used for these outcomes, and the results will be reported in a narrative and tabular form.

#### Sample Size

The aim of this study is to test the feasibility of *Pregnant Faster* and explore the barriers that might be encountered. On the basis of our previous experiences with including the described target group, we are confident that 40 participants will be sufficient to reach our aim.

### Ethics Approval

This study was assessed and approved by the Medical Ethical Committee of the Erasmus University Medical Center, Rotterdam, The Netherlands (MEC-2020-0974). Considering the low risk of this study, composing a Data Safety Monitoring Board was deemed unnecessary.

## Results

### The App Design

The name *Pregnant Faster* serves as a first microlevel nudge to join the study because most women who wish to have a baby often hope to become pregnant quickly. Using the app and its various components, all of which form microlevel nudges, will lead to earning so-called *coins.* Earning coins functions as a microlevel nudge because it stimulates participants to use the app.

**Figure 4:**
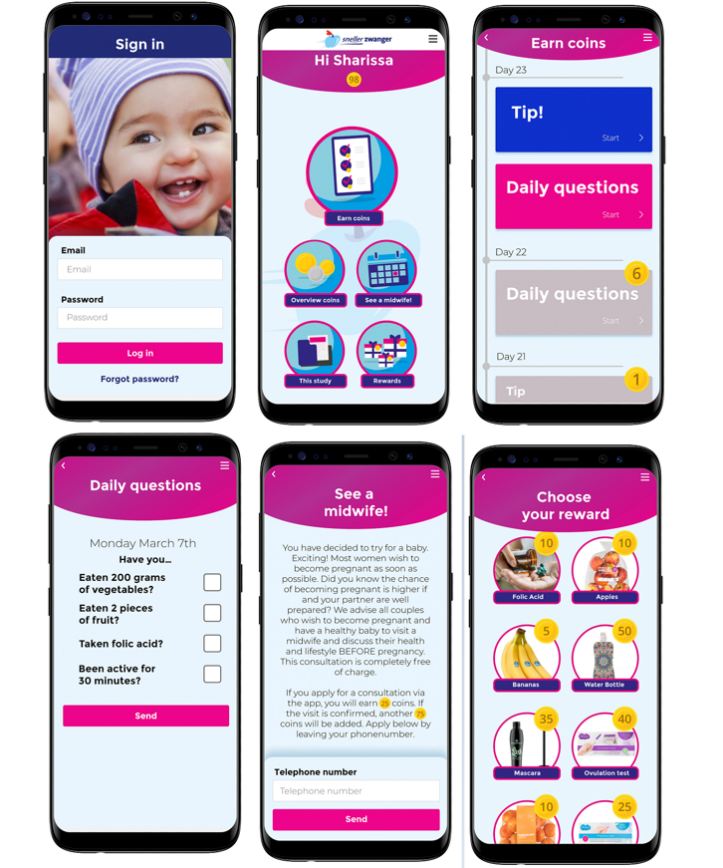
Screenshots of the mobile health app Pregnant Faster.

Participants log-in with their email address and a password that yields 1 coin per day. The first log-in yields 50 coins to immediately stimulate the participants to engage with the app. After log-in, a dashboard appears, containing five buttons: (1) Earn coins, (2) Overview coins, (3) See a midwife! (4) This study, and (5) Rewards (Figure 4).

Button 1, “Earn coins,” leads to a timeline in which new information appears daily in blogs or tips. Reading these yields 4 to 8 coins depending on the reading time and reading difficulty. Earning coins acts as a microlevel nudge to engage in reading relevant information about how to adequately prepare for pregnancy and ameliorate lifestyle and preconceptional health. Putting pregnancy preparation into the participant’s focus stimulates active thought on this topic, which in turn may lead to increased motivation for pregnancy preparation and healthier choices. In the same timeline, a daily questionnaire appears in which participants can tick off if they have eaten enough fruit and vegetables, exercised, and taken their folic acid supplements. Each ticked-off point yields 2 coins. This questionnaire also acts as a microlevel nudge because it serves as a reminder and motivator, which may lead to performing the behavior that can be ticked off.

Button 2, “Overview coins,” displays when and how coins have been earned and how many coins have been spent on which products. Having saved or spent a certain number of coins may nudge women to try and earn more coins, leading them back to the timeline in which they learn about properly preparing for pregnancy.

Button 3, “See a midwife!” contains information regarding what PCC entails and for whom it is meant. Here, we try to encourage participants to sign up for a consultation with a midwife in their vicinity and offer an easy way to sign up for a consultation in their neighborhood by filling in their phone number, which yields 25 coins immediately and an additional 75 coins after the visit has been confirmed. Minimizing the effort required to register for a PCC consultation serves as a microlevel nudge by lowering the threshold to engage in PCC.

Button 4, “This study,” contains information about the study itself, the researchers, and where to find additional information and contact details for questions, help, or complaints.

Button 5, “Rewards,” contains a list of rewards accompanied by images that participants can spend their coins on. Participants can choose from a variety of rewards such as folic acid supplements, fruits, nail polish, mascara, ovulation or pregnancy tests, newborn clothing, water bottles, and other healthy or fun rewards that may contribute to preconceptional health.

In our earlier ethical research and interview study [34], it became apparent that adequately respecting participants’ autonomy and preferences is imperative when it comes to increasing motivation for certain behaviors. Women wanted to have the option to either save up for a large reward or spend their coins on multiple smaller rewards. When participants choose their rewards freely, this will automatically lead them to choose the timing and type of reward that motivates them the most. In other words, being able to freely choose supports the app in stimulating women with different preferences regarding the timing and types of rewards to engage in pregnancy preparation and visit a PCC consultation. To satisfy this requirement, the app contains multiple types of rewards of varying value.

Another microlevel nudge lies in the varying value of the coins. One coin represents a monetary value of €0.06 to €0.26 (US $0.06 to US $0.26). By making healthy rewards, such as folic acid supplements and fruits, relatively cheap, we steer participants toward picking healthy products over luxury goods, while still allowing them to choose luxury goods if that is what stimulates them most to earn coins and engage in PCC. For instance, the mascara and folic acid supplements are of equal monetary value and cost €3.00 (US $3.00). However, the folic acid supplements can be ordered for 10 coins whereas ordering the mascara will cost the participant 35 coins.

Participants of our previously conducted interview study [34] mentioned that it might be painful for them if they could only choose pregnancy and baby-related products, in case of not getting pregnant quickly. To take this into account, we have included rewards in the app that are not relatable to pregnancy or babies, such as water bottles, a book voucher, and resistance bands for physical exercise.

The design of *Pregnant Faster* makes the app suitable for all women because it automatically matches participants’ personal preferences and their current level of motivation to prepare for pregnancy and engage in PCC. This is done by allowing participants to freely choose the type and timing of their rewards and by matching their current “Stage of Change,” as described by Prochaska and DiClemente [39].

**Figure 5:**
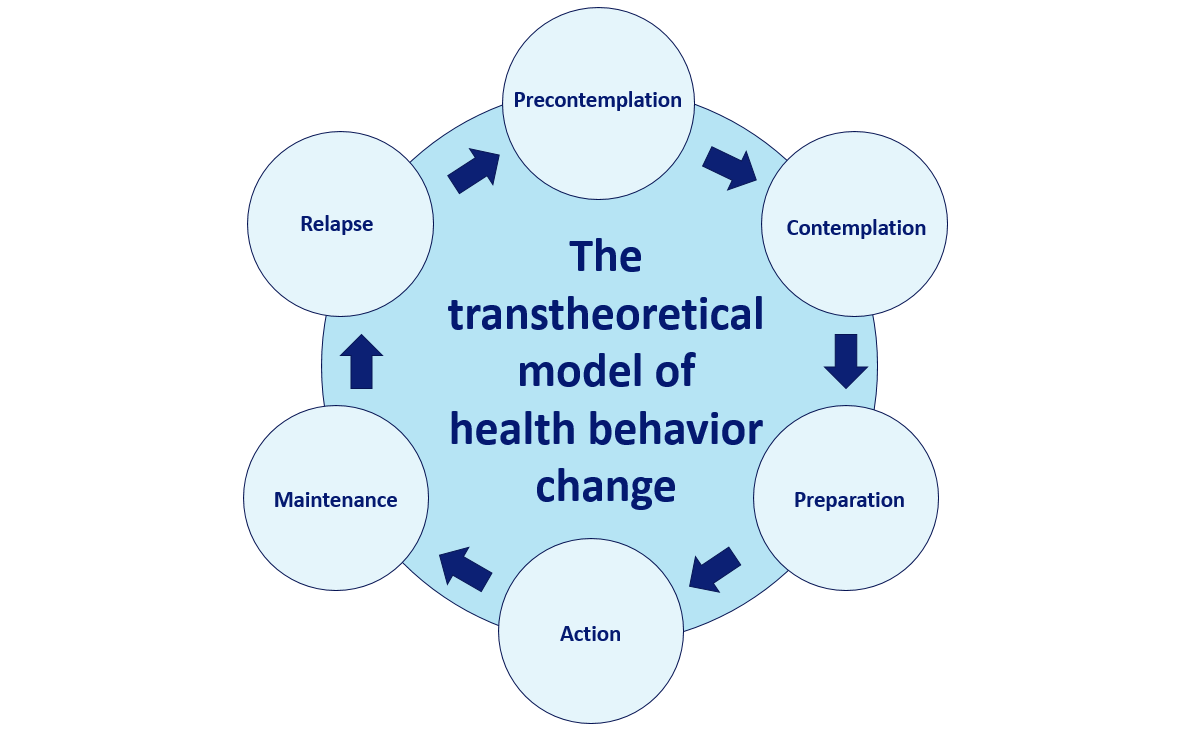
The transtheoretical model of health behavior change by Prochaska and DiClemente [36]. (1) Precontemplation: unaware of the need for change and unwilling to change. (2) Contemplation: aware of the problem but not yet committed to change. (3) Preparation: believes change is good, intent on taking action, and planning to start. (4) Action: actively changing behavior or creating new habits. (5) Maintenance: behavior has changed, committed, and resolved; must be aware of the possibility of relapse. (6) Relapse: relapsing into old behavior; may skip precontemplation in the next cycle.

The transtheoretical model of behavior change by Prochaska and DiClemente [39] describes the different and subsequent stages of changing a certain behavior: precontemplation, contemplation, preparation, action, maintenance, and relapse (Figure 5). Each stage requires a different type of approach [40]. For instance, if a woman has an unhealthy diet and is currently in the precontemplation stage, is not aware of a problem, and is not willing to change, helping her set up a detailed healthy eating plan may lead to her feeling overwhelmed or rejecting the idea that a healthy diet is important, which results in resistance and noncompliance. The first step for this specific woman would be to help her discover why a healthy diet is important to her and which benefits it could possibly yield for her. This will help her acknowledge that her current diet may be problematic and requires a change: the contemplation stage. However, if the woman has been contemplating improving her diet for a while, helping her set up a practical plan may be the exact nudge she needs to help her reach the next stages of change: preparation and action. The choice to use this model was based on the fact that it is widely used and taught in medical education and health care professions, as it provides solid instructions on how best to approach the patient or client to encourage and support them in changing unhealthy behaviors, in addition to the available evidence regarding its effectiveness [41,42] and the amount of experience of the authors in the application of this model.

It is clear that the most effective approach differs by stage and that the current stage of change is very personal. However, we chose not to assess the participants’ current stage of change to minimize what participants could deem *fruitless engagement*—spending time in the app without being rewarded for it, which could diminish interest in the app. Therefore, *Pregnant Faster* was designed to passively match all possible stages, as described by Prochaska and DiClemente [39]. If a participant is currently not preparing for pregnancy and is not interested in doing so (precontemplation), it is still attractive to read the information *Pregnant Faster* offers because of the coins she can earn and spend on rewards to her liking. The blogs and tips in *Pregnant Faster* focus on conveying that healthy choices and PCC may help to have a healthy baby sooner, a goal that many women strive for. This may cause a participant to start contemplating if she too could get pregnant faster by actively preparing for pregnancy and visiting a midwife for PCC. She might even start to plan how she could take action (preparation). If a participant is already preparing for pregnancy by, for instance, eating more vegetables (action or maintenance), reading about the benefits of this behavior in the timeline of the app will act as a stimulant to maintain this behavior or maybe even make a plan to also eat 2 pieces of fruit per day (preparation). For women who already have a healthy diet and lifestyle, and are currently in the maintenance stage, the app will serve as a stimulant to sustain healthy behavior and may help to prevent a relapse (maintenance). In conclusion, the app appeals to women in each stage of change by either encouraging new healthy behavior or the continuation of preexisting healthy behavior.

Participants in any stage of change may choose to register for a PCC consultation as the reward is high and the required action (filling in their phone number) is relatively small. This was decided consciously to meet the main objective of *Pregnant Faster*, stimulating pregnancy preparation by visiting a PCC consultation provided by a health care provider. These face-to-face consultations allow health care providers to directly apply motivational conversation techniques [43] and actively match the participant’s current stage of change on the topics that are most relevant for them, for instance, smoking cessation, an unhealthy diet, or a sedentary lifestyle. Having an engaging, live conversation in which the participant can ask questions and feels heard and supported may be the greatest encouragement or nudge to engage in adequate pregnancy preparation and make healthy choices. Furthermore, a live consultation allows for relevant referrals (eg, for smoking cessation) without relying on the participant to take the first step.

This intervention is completed after 4 weeks. If women still have coins left by the end of the intervention, they will be able to exchange these for rewards within 2 weeks after the completion date or 2 weeks after their PCC consultation. The app and timeline will continue to be available for participants, so they can access its information and register for a PCC consultation whenever they please, but they will no longer be able to earn or spend coins by using the app.

The iterative developmental process has led to the detailed design of the app *Pregnant Faster*, which we have built in cooperation with the event agency Improve in Delft, the Netherlands, and the mobile app manufacturer TJIP The Platform Engineer. *Pregnant Faster* is an app of 5.2 MB and is currently only available in the research setting for Android by a download link and for iPhone through the Apple store. The app has a modern, colorful interface (Figure 4) inspired by the preferences of the target group. Overall, the app was designed to fit the user’s needs and preferences concerning the content of the app as well as easy and intuitive operating.

### Cohort Study

Results are expected to be published by December 2023.

## Discussion

### Principal Findings

In this paper, we have described the iterative cocreation design process of the app *Pregnant Faster*, an mHealth app–based nudge specifically designed for women with a vulnerable health status based on their SES who have a higher risk of adverse pregnancy outcomes. In addition, we have described the protocol of the cohort study aimed at establishing the feasibility of using *Pregnant Faster* to encourage these women, in an ethically justified way, to adequately prepare for pregnancy by engaging in PCC and making healthy nutrition and lifestyle choices.

In this proposed study, women will be nudged through an app that rewards them to encourage them to make healthy choices. The nature of an incentive-based intervention, or nudge, is such that its aim is to *influence* the participant’s behavior. There is a fine line to tread between nudging and manipulation, which we consider in this protocol. Nudging entails that alternative choices are never taken away or obstructed, and choosing differently does not require significant extra effort or create a great sense of loss, as this would count as coercion. This study does not force women to prepare for pregnancy or engage in PCC. Using the app or registering for a consultation is optional, and the offered rewards are of such value that they will not coerce participants into actions they do not value or wish to perform.

Furthermore, the aim of the nudge must be beneficial to the target group and must align with their own preferences and aims. In this case, the intervention aimed to adequately prepare women with a vulnerable health status for pregnancy to reduce the chance of adverse pregnancy outcomes. This aligns with the preferences and aims of the participants as they want to give birth to a healthy baby. For manipulation to be prevented, transparency concerning the aim of this study is crucial. Therefore, proper explanation, obtaining informed consent, and ensuring that the participant consents to being influenced is of utmost importance.

van der Windt et al [22] established that blended care is an effective method to improve lifestyle and nutrition choices. In this study, we will use an mHealth app to offer an intervention that aims to encourage women to learn about healthy pregnancy preparation via an app and visit a face-to-face PCC consultation. In other words, it aims to provide blended care while still offering important information to women who are not yet ready to engage in a face-to-face consultation.

The use of smartphones is widespread, even among women with a low SES [44]. This means that *Pregnant Faster* as an app-based nudge will be widely available and easily accessible for the target group, allowing them to use the app privately and at a time that suits them best, which reduces the burden of participating in the study. The attractive interface and overall positive outlook applied in the app are aimed at further reducing any negative feelings that may result from learning about necessary lifestyle behavioral changes, resulting in an app that is fun to have and use. A foreseen possible limitation of the app design lies in the fact that it cannot yet be altered to best suit the participant’s personal situation regarding her gestational age or how long she has been trying to become pregnant. However, even though *Pregnant Faster* does not specifically target women with difficulty conceiving, it does address the experiences associated with subfertility, which means that this group may also benefit from using the app. For example, the offered information aligns with the nutrition and lifestyle advice given to couples in the fertility outpatient clinic of the University Medical Center where this study has taken place. Furthermore, attention is paid to which factors influence the chance to become pregnant and have an ongoing pregnancy, specifying which factors are within and outside one’s own control. By providing clarity on what can and cannot be influenced, including the size of the expected effects, we aim to encourage healthy lifestyle behavior without possibly burdening participants with the idea that they themselves would be responsible for (possible) subfertility or miscarriage cessation. Furthermore, within the blogs and tips, advice is given on when and how to find support when trying for a baby takes longer than expected, considering women’s age and medical history in line with Dutch General Practitioner guidelines on referral for fertility treatments. In addition, the stress that may accompany trying to get pregnant is acknowledged and tips are provided to cope with these experiences.

With regard to the study design, the outcomes comprising *Pregnant Faster’s* feasibility allow for practical evaluation, which will form solid stepping stones to further develop the app. The design of the app is such that we aim to continue its development throughout the coming years and possibly explore its use in different target groups, with and without rewards. A possible limitation in the study design pertains to the interviews with the first 10 participants that have been used to develop the additional questionnaire on user experiences. To evaluate the group of 40 women as one, these interviews will be used to fill out the developed questionnaire. It might be more prudent to qualitatively analyze these interviews and view these 10 participants as a separate group. However, as this feasibility study mainly aims to provide holdfasts for further development of *Pregnant Faster*, we believe that this method will suffice.

A potential risk of using *Pregnant Faster* may be that providing rewards can “crowd out” intrinsic motivation to make healthy choices [45,46]. In other words, the healthy behavior that women value and display before the start of the intervention, diminishes or vanishes after the intervention ends, and rewards are no longer provided. For example, if women went to the gym before the intervention, they could maintain this habit and receive rewards for it. This influences the way they view and value this behavior by shifting their focus from health-related rewards, such as strength and fitness, to material rewards. When the intervention ends and going to the gym no longer leads to material rewards, these women might be less inclined to exercise than they were before they participated in the intervention. This is caused by increasing extrinsic motivation for a behavior at the expense of intrinsic motivation.

If a nudge only appeals to extrinsic motivation, the healthy behavior “dies out” as soon as the intervention ends [46]. However, scaffolding intrinsic motivation leads to a more sustainable change because it focuses on health-related rewards. Overall, women who want to become pregnant wish to have a healthy baby. Educating them about the control they may exercise on their pregnancy outcomes by taking up PCC and making healthy nutrition and lifestyle choices fits their wish to have a healthy baby and may increase their intrinsic motivation to make healthy choices. This cancels out the *crowding out* effect and allows for continuation of healthy behavior because the focus is not placed on limited material rewards but on continued health-related rewards, making the behavioral changes more sustainable.

Another potential risk lies in how the app makes women feel. It is known that mHealth apps may evoke feelings of guilt, shame, and stress and even discouragement when participants fail to meet the app’s goals [47]. It is paramount that the tips and blogs in *Pregnant Faster* convey that small steps are worth celebrating and that women are not obliged to strive for a perfect lifestyle to have a healthy baby. Furthermore, the app must also contain information on the fact that a healthy lifestyle increases the chance of a healthy baby but offers no guarantees. It must be stated that not all adverse outcomes can be prevented and that an adverse pregnancy outcome does not mean that the woman (or her partner) is to blame.

When this pilot study has been conducted, we expect to have obtained valuable information on how to further adjust *Pregnant Faster* to best fit the needs of women with a vulnerable health status and encourage PCC uptake in this group. In other words, this proposed study can be viewed as yet another phase of the iterative process, and the outcomes will be used to update and alter the app to further develop the design of *Pregnant Faster*. Furthermore, practical information on, for example, the number of registrations of eligible and noneligible women will provide guidance on our methods to reach the target group and evaluation of the rewards, and the frequency of allocation will provide more insight into which rewards are deemed most desirable. Finally, any barriers encountered during this pilot study will be assessed to avoid such barriers in the future.

### Conclusions

We have developed an mHealth app (*Pregnant Faster*)–based nudge that aims to encourage women with a vulnerable health status to engage in pregnancy preparation by taking up PCC, to improve the high incidence of adverse pregnancy outcomes in this group.

The risks and burdens of participating in this study will be minimal, whereas the benefits for both the mother and (unborn) child are potentially substantial. Furthermore, we designed a study protocol to establish the feasibility of *Pregnant Faster*. If the results of this study indicate that using *Pregnant Faster* to increase the uptake of PCC in women with a vulnerable health status is feasible, we will implement the obtained insights and further develop the app. For example, if a specific barrier in joining the study or registering for a PCC consultation is encountered, we will attempt to overcome these barriers to improve inclusion rates and increase PCC consultation requests and continue this study with an updated version of the app and an additional 400 inclusions, thus attempting to establish the (cost) effectiveness of *Pregnant Faster* with regard to increasing the uptake of PCC.

Finally, we aim to investigate the possibility of offering *Pregnant Faster* without rewards to all women who wish to become pregnant, regardless of their health status. Earning material rewards forms just one of the multiple microlevel nudges in this macrolevel nudge and is meant to attract women with little knowledge of, or headspace or motivation for, pregnancy preparation. However, many women look for information on how to become pregnant quickly and healthily. For these women, the knowledge and motivation provided by *Pregnant Faster* may also be valuable and help them adequately prepare for pregnancy.

## Appendix

Multimedia Appendix 1 The mHealth App Usability Questionnaire.

Multimedia Appendix 2 Topic list—user experiences Pregnant Faster.

## Abbreviations

MAUQ: mHealth App Usability Questionnaire
mHealth: mobile health
PCC: preconception care
SES: socioeconomic status

## Data Availability

As this paper concerns a design process and proposed study, no pilot study data are available to share as yet. However, the design process of *Pregnant Faster* and the proposed pilot study are based on earlier research conducted by this group. Articles about *Smarter Pregnancy* and its development and testing have been published previously [19,26], as has the interview study on the preferences of susceptible women regarding a loyalty program [34]. Due to the ethical framework currently being submitted, we are unable to provide its data, as that would be in conflict with the conditions of submission. Further inquiry with regard to the ethical framework and reports of the strategic work session (which are written in Dutch) can be provided by the authors at reasonable request.
