# Supplementary material for: Stimulating the Uptake of Preconception Care by Women With a Vulnerable Health Status Through mHealth App–Based Nudging (Pregnant Faster): Cocreation Design and Protocol for a Cohort Study
Source: JMIR Res Protoc. 2023 Aug 9;12:e45293. doi: 10.2196/45293 (PMC10448288; doi:10.2196/45293)
Supplement: Multimedia Appendix 1 [file resprot_v12i1e45293_app1.docx]

**The mHealth Usability Questionnaire – *Pregnant Faster***

Study Identification Number

Submission date

Answer options

I strongly agree

I agree

I slightly agree

Neutral

I slightly disagree

I disagree

I strongly disagree

Questions

The app was easy to use.

It was easy for me to learn how to use the app.

Navigation was consistent when switching between screens.

The interface of the app allowed me to use all the features the app offers (e.g. enter information, respond to reminders, view information).

Whenever I made a mistake with the app, I was able to fix it easily and quickly.

I like the interface of the app.

The information in the app was well organized so that I could easily find the information I needed.

The app provided information to let me know the progress of my action.

I feel comfortable using this app around other poeple.

The amount of time it takes to use this app agrees with me.

I would use this app again.

Overall, I am satisfied with this app.

I find this app useful for my health and well-being.

This app improved my access to healthcare.

The app has helped me to effectively take control of my health.

This app has all the features and capabilities I expected it to have.

I was able to use the app even when the internet connection was poor or unavailable.

This app provides an acceptable way to receive health care, such as access to educational materials, tracking my own activities, and conducting self-assessments.

Do you have any comments or comments about the app? [open question]
