# Supplementary material for: Stimulating the Uptake of Preconception Care by Women With a Vulnerable Health Status Through mHealth App–Based Nudging (Pregnant Faster): Cocreation Design and Protocol for a Cohort Study
Source: JMIR Res Protoc. 2023 Aug 9;12:e45293. doi: 10.2196/45293 (PMC10448288; doi:10.2196/45293)
Supplement: Multimedia Appendix 2 [file resprot_v12i1e45293_app2.doc]

Topic List – user experiences Pregnant Faster

Grand tour

Could you please describe how you have experienced the past four weeks using the app?

1. **Overall experience**
   1. Positive / Negative
   2. Useful / Not useful
   3. Experienced intensiveness of the intervention
   4. Reasons for frequent / infrequent use
2. **Participants experiences regarding the inclusion procedure**
   1. Level of necessary effort
   2. Level of difficulty
   3. Guidance received from the researchers
3. **Interface and functionality of the app**
   1. Participants type of phone and operating system
   2. Positive and negative comments
   3. Additional wishes and recommendations.
   4. Comments on log-in flow
4. **Coins and rewards**
   1. Experiences regarding earning and saving coins
   2. Comments on received rewards
   3. Comments on the available rewards
   4. Comments on logistics such as delivery time and manner of delivery
   5. Additional wishes and recommendations
5. **Workflow registering for PCC consultation**
   1. Level of necessary effort
   2. Level of difficulty
   3. Guidance received from the researchers
   4. Reason for registering or not registering for a consultation
6. **Experiences with regard tot the PCC consultation**
   1. Expectations prior to the consultation
   2. Level of satisfaction regarding the consultation
   3. Overall opinion on the consultation
7. **Self reported behavior change during and after the intervention**
